# Optimus Primer: A PCR enrichment primer design program for next-generation sequencing of human exonic regions

**DOI:** 10.1186/1756-0500-3-185

**Published:** 2010-07-07

**Authors:** Andrew MK Brown, Ken Sin Lo, Paul Guelpa, Mélissa Beaudoin, John D Rioux, Jean-Claude Tardif, Michael S Phillips, Guillaume Lettre

**Affiliations:** 1 Beaulieu-Saucier Université de Montréal Pharmacogenomics Centre, Montreal, Quebec, Canada; 2 Université de Montréal, Montreal, Quebec, Canada; 3 Montreal Heart Institute Research Center, 5000 rue Bélanger Est. Montreal, Quebec, H1T 1C8 Canada

## Abstract

**Background:**

Polymerase chain reaction (PCR) remains a simple, flexible, and inexpensive method for enriching genomic regions of interest for next-generation sequencing. In order to utilize PCR in this context, a major challenge facing researchers is how to generate a very large number of functional PCR primers that will successfully generate useable amplicons. For instance, in an exon-only re-sequencing project targeting 100 genes, each with 10 exons, 1,000 pairs of primers are required. In fact, the reality is often more complex as each gene might have several isoforms and large exons need to be divided to maintain the desired amplicon size. With only a list of gene names, our program Optimus Primer (OP) automatically takes into account all these variables, and can generate primers with no need to provide genome coordinates. More importantly however, OP, unlike other primer design programs, uniquely utilizes Primer3 in an iterative manner that allows the user to progressively design up to four iterations of primer designs. Through a single interface, the user can specify up to four different design parameters with different stringencies, thus increasing the probability that a functional PCR primer pair will be designed for all regions of interest in a single pass of the pipeline.

**Findings:**

To demonstrate the effectiveness of the program, we designed PCR primers against 77 genes located in loci associated with ulcerative colitis as part of a candidate gene re-sequencing experiment. We achieved an experimental success rate of 93% or 472 out of 508 amplicons spanning the exonic regions of the 77 genes. Moreover, by automatically passing amplicons that failed primer design through three additional iterations of design parameters, we achieved an additional 170 successful primer pairs or 34% more in a single pass of OP than by conventional methods.

**Conclusion:**

With only a gene list and PCR parameters, a user can produce hundreds of PCR primer designs for regions of interest with a high probability of success in a very short amount of time. Optimus Primer is an essential tool for researchers who want to pursue PCR-based enrichment strategies for next-generation re-sequencing applications. The program can be accessed via website at http://op.pgx.ca.

## Findings

The development of next-generation sequencing (NGS) technologies has dramatically increased the size and scale of sequencing experiments. It is now possible to produce several gigabases of DNA sequence in a short period of time [1]. To date, the cost of whole human genome sequencing remains prohibitive. Focusing NGS experiments to specific genomic regions is an alternative, cost-effective approach to whole genome sequencing but it requires the enrichment of the targeted regions before library construction. Several hybridization-based methods - each with its own strengths and weaknesses - have been developed [2]. While these DNA enrichment methods continue to be developed and improved, a simple and inexpensive alternative is PCR. PCR is a robust, well-understood, very accessible and flexible strategy for DNA enrichment. It also allows for the very specific amplification of targeted regions without the high background found in hybridization-based methods. Hundreds or even thousands of PCR amplicons that span selected genomic regions of interest can be pooled together and used as input material for the sequencing reaction. PCR has been traditionally used for classic Sanger chemistry-based sequencing of few genes. However, the throughput of next-generation DNA re-sequencing is such that new tools need to be developed to facilitate the implementation of PCR as enrichment strategy for these new sequencing methods.

Enrichment of exonic regions is of particular interest as the functionality of variations within these regions can be more easily inferred than variations in non-coding DNA. Surveying genetic variation by NGS for all exons in candidate genes, such as those identified in genome wide association studies (GWAS), may contribute to the identification of the causal genes and variants, and therefore the underlying biology of the disease.

**Figure 1:**
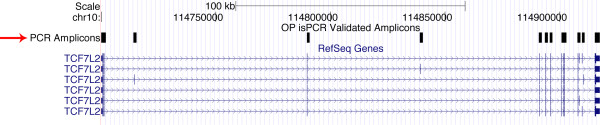
**Example of Exon Coverage of Optimus Primer Designed Amplicons**. Image from the UCSC genome browser showing Optimus Primer design results for the gene TCF7L2 [10]. The black boxes (highlighted by the red arrow) indicate the positions of all isPCR validated amplicons designed by OP. Note that only the gene name, TCF7L2, was submitted into the pipeline yet amplicons were designed spanning the exons (indicated by blue boxed) for all 6 known isoforms of the gene.

In order to utilize PCR in the context of NGS, where hundreds of genes are resequencd, a major challenge facing researchers is how to generate a very large number of functional PCR primers that will successfully generate useable amplicons. For instance, in order to target the re-sequencing of 100 genes, each with 10 exons, this requires 1,000 pairs of PCR primers. In fact, the reality is often more complex as each gene might have several isoforms and the enrichment strategy must accommodate large exons that require overlapping PCR products to maintain the desired amplicon size. Thus, to address this significant bioinformatic need in setting up robust NGS enrichment pipelines, we have developed the Optimus Primer (OP) website http://op.pgx.ca where users can import a gene list with no need to provide genome coordinates to generate a complete list of PCR primer pairs (see figure 1 and table 1 for example). Other groups have developed pipelines to facilitate PCR primer design, [3-5]. These programs often cannot accommodate primer design from the gene names alone and require significant background research to obtain sequence information. Furthermore, most of these programs are only capable of processing a limited list of genes at a time and lastly, all of these programs do not possess the ability to handle multiple design criteria to design multiple iterations of the same amplicon in a single run, thus maximizing the likelihood that a successful primer pair will be designed. Therefore these algorithms are not suitable for the large number of PCR primers required for NGS enrichment. In contrast, OP is able to handle many genes simultaneously and requires only the gene name as input. The user of the program has the ability to set the design criteria for up to four iterations of primer designing parameters that allows the user to progressively modify the design iterations to make them less stringent in order to design primers for all submitted genes or regions. This makes this program especially useful for the rapid design of the large number of primers needed in targeted resequencing experiments.

**Table 1:** Example PCR Primer Pairs Designed After Submitting TCF7L2

Region Name	Forward Primer	Reverse Primer
TCF7L2_chr10_exon1-3_1	CTGTTTATTTATGCACACGTCACTG	gTACTCACCTTCTTCCAAACTTTCC

TCF7L2_chr10_exon1-3_2	GATTCTTTTTCTCCCCCTTCTC	AGCCAGCAATCTCCACTAGAAAG

TCF7L2_chr10_exon4_1	TCCCTGTATGCTTATTGAATAGGTG	CCCAGAAAAGAAGTCATGGAATAAC

TCF7L2_chr10_exon5_1	GGCTAGTTGTTCTGCATTTACTTTC	AGCTCCAAAATAAGGAGGCAGTTAG

TCF7L2_chr10_exon6_1	AATAGGAGAGTTCTGATTTGTGCTG	GACTTATTCCACATTTTGTCTCTCC

TCF7L2_chr10_exon7_1	CATCTTAGAAAATCCAGGTGAGAGG	AACAACTGGGATAAAAAGGGGATG

TCF7L2_chr10_exon8_1	ATGAGATGAAACCTACCAACAACTC	TGTTGTTCAGAGTACAGATCACTGG

TCF7L2_chr10_exon9_1	CTTACTGTGCAGAGAGAACTTTTCC	ATTAGCGACTAAAACATACTGCTGC

TCF7L2_chr10_exon10-12_1	ACGATTTACACAGCTTTCTGTCTTC	CTATGTCATTCTGTCATTTGCTCAC

TCF7L2_chr10_exon10-12_2	CTGAGTGCACGTTGAAAGAAAG	AGAGAGGACTAGGCAGATCCTGTAG

TCF7L2_chr10_exon13-14_1	CAGACACTCTTCTCACATCTGTTTC	GGAGGTTTATTACTGAATTCCTTCC

TCF7L2_chr10_exon15-16_1	CTCGCTTCTCTCTTGAACTCATTC	CTTCTCCATGTGTCTCGACTCTAAG

TCF7L2_chr10_exon17_1	TATTCACAGATAACTCTCTCCCCTG	TCTATTAAGTGTTGAGTAGCGTCCC

TCF7L2_chr10_exon17_2	ACATCTGGTTTTTAAACCGTAAGGG	GAGCATAAAACGGAACAGTAACATC

TCF7L2_chr10_exon17_3	CATATTACATACGAGTAGGCAGCAG	CGATACTGTGGTCACCTTAAGAAAC

After having only submitted the gene name (TCF7L2) Optimus Primer was able to successfully design PCR primers to amplify all exons (see figure 1 for amplicons position). Note that exon in close proximity to each other were merged into single amplicons (exons 1-3 for example) and that large exons were divided into several amplicons to maintain consistent amplicons size (exon 17 for example).

## Implementation

Optimus Primer (OP) is a web-based automated pipeline that requires the user to submit only a gene list, or list of regions of interest, and primer design parameters. The pipeline consists of four steps. First, all exons for all known isoforms for each gene submitted are identified using the RefSeq database [6]. This step is skipped if regions of interest are submitted. A list of all unique exons for each gene is then generated with exons/regions that are in close proximity to each other (< 25 base pairs for example) merged into a single element in the list. Second, the pipeline extracts the desired genomic sequences from the current build of the human genome (currently hg18/NCBI36), plus additional flanking sequence at a length defined by the user to facilitate the design of the PCR primers. OP will prioritize the design of the primers to these flanking regions to ensure complete coverage of the specific exonic regions. The user has the option of including or excluding sequence that has been masked with RepeatMasker [7]. Additionally, polymorphisms from the current build of dbSNP (currently build 130) can be masked to ensure that primers are not designed to locations with underlying SNPs [8]. Primer3 has been integrated into the pipeline to design PCR primers using user defined parameters [9]. Exons/regions that are larger than the specified amplicon size will be automatically split into smaller amplicons, with a minimum 25 bp overlap to ensure that every base can be amplified and sequenced. Exons for which no PCR primer design is possible using the initial parameters are passed on to a second iteration of Primer3 with modified design criteria defined by the user.

Currently, the pipeline allows the user to define up to four iterations of Primer3 design criteria in a single pass to attempt to design PCR primers for all amplicons with up to 5 primer pairs for each amplicon. The final step of the pipeline is to run all designed PCR primers through the UCSC Genome Browser in-silico PCR (isPCR) utility as a validation step for the primer pairs selected [10]. The isPCR utility allows the user to check the human genome for the presence of unique primer pairs, ensures that they are designed correctly on opposite strands, that they are the correct distance apart and generates a report of the theoretical amplicons produced by the primer pair. OP then uses this data to generate a report for all primer designs as well as the percent coverage for each exon/region for each gene for all isPCR validated primer pairs. Primers designed with OP can then be used to amplify genes of interest as the enrichment step prior to library construction for NGS experiments. In particular, because PCR is flexible and easily implementable, OP will be ideal to target for NGS genes that are difficult to enrich using solid- or liquid-based capture reagents and for genes that are very polymorphic. Additionally, for genes whose annotation is dynamic from one build of the human genome to the other, PCR can be easily adapted whereas probes-based capture reagents will need to be re-synthesized.

## Results

As part of a high-throughput DNA re-sequencing project to identify genetic risk factors for ulcerative colitis, we targeted 77 genes for NGS. PCR amplification was selected as enrichment strategy and we opted for pooled sequencing. This corresponds to 867 unique exons and a total of 237 kb of sequence. OP divided the exons into 993 amplicons (average size of 586 bp). After a single pass of OP, primer pairs were designed for 861 (87%) of amplicons using our stringent design parameters (see table 2). PCR primers could not be designed for all desired amplicons for reasons such as low complexity DNA and underlying variation. However with additional effort, it is possible to modify (loosen) Primer3 parameters to allow for the design of PCR primers in these regions. 714 of the OP designed primer designs (861) passed our isPCR evaluation and validation process (representing 72.5% of desired exons). In the lab, we tested 508 of the isPCR validated primer pairs using 7 different genomic DNA samples. We demonstrated successful PCR products for 472 amplicons (92.9% success rate) using agarose gel electrophoresis and/or PicoGreen quantification. Of the 508 primer pairs tested, 338, 18, 80 and 72 were designed in the first, second, third and fourth iterations of Primer3 respectively. By including three additional design iterations with different design parameters with different stringencies, it resulted in an additional 170 successful primer pairs or an increase of 34% more designs in a single pass of OP than by conventional methods. The success rates were 93%, 84%, 99% and 97% for the four iterations. Therefore, we feel that our approach of using multiple design iterations with progressively looser design parameters is a valid approach to successful primer design. In our specific experiment, amplicons were pooled and libraries were constructed according to Illumina's recommendations. We re-sequenced the targeted exons using a single-end 36-base pair protocol on our Genome Analyzer II. We generated 4.5 gigabases of DNA sequences, 89% of which was on target. This allowed us to generate 40× coverage and identify over 700 coding DNA sequence variants. Specific details from genetic risk factors for UC will be presented elsewhere.

**Table 2:** Primer Design Parameters Used in the Four Passes of Primer3.

	First Pass	Second Pass	Third Pass	Fourth Pass
**Optimum Size (BP)**	25	25	25	25

**Minimum Size (BP)**	20	20	18	20

**Maximum Size (BP)**	30	30	33	30

**OptimumTM (°C)**	60	60	60	60

**Minimum TM (°C)**	55	55	50	55

**Maximum TM (°C)**	65	65	70	65

**Optimum GC%**	50	50	50	50

**Minimum GC%**	40	40	35	40

**Maximum GC%**	60	60	65	60

**GC CLAMP**	Yes	No	No	Yes

**NUM RETURN**	1	1	1	1

**PRODUCT SIZE RANGE (BP)**	400-600	400-600	400-600	400-600

**Repeat Masking**	Yes	Yes	Yes	No

**SNP Masking**	Yes	Yes	Yes	Yes

Optimus Primer allows the user to specify up to four sets of PCR primer design parameter simultaneously. Amplicons for which no primers are designed in the first pass are automatically passed on to the next pass. Above are the four sets parameters used to design primers in our validation experiments.

## Conclusion

PCR is currently the cheapest, simplest, most flexible approach for sample enrichment prior to NGS experiments. It also has some distinctive advantages over the less specific enrichment methodologies currently used for targeted next generation resequencing. In order to capitalize on PCR-based methodologies, hundreds if not thousands of PCR primers need to be designed. To address this gap in bioinformatic tools, we have developed Optimus Primer (OP), a web-based automated PCR design pipeline that facilitates the simultaneous design of PCR primers for the enrichment of exonic regions in multiple genes. This tool can be useful not only for the enrichment of exonic regions for NGS experiments, but it also has much more general applicability to other experiments that require the rapid design of PCR primers for multiple regions of interest such as genotyping, Sanger sequencing and real time PCR. With only a gene list and PCR parameters, a user can design hundreds of PCR primers in a very short timeframe.

## Competing interests

The authors declare that they have no competing interests.

## Authors' contributions

AMKB, KSL and PG wrote all software, created the website and drafted the manuscript. MB performed the PCR and validation experiments. GL conceived the project and helped draft manuscript. J-CT, MSP and JDR participated with the design and coordination of the project, and helped draft the manuscript. All authors read and approved the final manuscript.
